# Illuminating the hidden cost: A systematic review of cognitive late effects regarding cancer-related fatigue in treated paediatric brain tumors

**DOI:** 10.1016/j.tipsro.2024.100291

**Published:** 2024-12-09

**Authors:** Ingrid Vethe Hernes, Amalie Jansdatter, Anita Nordsteien, Mathilde Haraldsen Normann

**Affiliations:** a Haukeland University Hospital, Norway; b University Hospital of North-Norway, Norway; c University of South-Eastern, Faculty of Health and Social Sciences, Norway

**Keywords:** Radiation therapy, Radiotherapy, Paediatric brain cancer, Toxicity, Late effect, Fatigue

## Abstract

**Objective:**

Globally, in 2022, 30,871 children were diagnosed with CNS-tumors. Many have been treated with radiotherapy, and a significant number suffer from chronic late effects, including fatigue. This study aims to investigate previous research on the impact of cancer-related fatigue for neurocognitive function that can be related to radiotherapy in patients who have undergone primary brain radiotherapy before the age of 18.

**Methods:**

Conducted under PRISMA-S framework, this systematic review searched MEDLINE ALL (Ovid), EMBASE (Ovid), CINAHL (EBSCO), and PsycINFO (Ovid) for relevant studies. Criteria for inclusion were children under 18 who underwent radiotherapy for primary brain cancer, focusing on late cognitive side effects, published 2000–2023.

**Results:**

From 4,067 records, 10 studies were included, examining Proton Radiation Therapy (n = 4), X-ray Radiation Therapy (n = 3), and their comparisons (n = 3). The studies used various cognitive tests, and late effects that emerged were neurocognitive functions and disorders, intellectual functioning, specific cognitive functions and daily life, social functioning, and performance. These themes can be encompassed by cancer-related fatigue.

**Conclusions:**

The findings underscore critical need for more in-depth research to understand the health perception variations among children post-primary brain radiotherapy. Furthermore, detailed insights of treatment specifics, disease progression, target volume sizes, and doses to surrounding organs at risk are imperative.

## Highlights

• This literature review identifies four themes related to cancer related fatigue after primary brain radiotherapy given <18 years: 1) neurocognitive functions and disorders, 2) intellectual functioning, 3) specific cognitive functions and 4) daily life, social functioning, and performance. In addition, three studies, not including information about the dosage of the radiotherapy given are highlighted due to the information they are providing about CrF.
• This literature review findings reveal variations in cognitive outcomes after radiotherapy, with both proton therapy (PRT) and photon therapy (XRT) showing distinct effects.
• The results presented in this study emphasize the wide range of cognitive challenges these children face. This indicates that although some children show signs of improvement in performance status over time, there are clear cognitive and social challenges that affect their ability to function optimally in everyday and social settings.

## Introduction

Globally in 2022, 275,713 new cases of all types of cancer occurred among children and adolescents aged 0–19 years. Of these, 30,871 emerged in the central nervous system (CNS), including the brain, across all countries for the same age group [1]. The treatment of primary brain cancer is usually multi-modality. During the period of 2019–2022, 79.6 % had surgical interventions, 27.3 % had chemotherapy and 23.4 % radiotherapy. Radiotherapy plays an important role in treatment of paediatric brain tumors [2] and the radiotherapy dose and fractionation varies based on histological classifications [3], [4].

Children and adolescents in need of radiation treatment are at risk of potential lifelong late effects and need long-term follow-up [5], [6], [7], [8]. Almost 80 % of children and adolescents who receive a cancer diagnosis become long-term survivors, and over 60 % reported having at least one chronic late effect [9]. Children who receive radiotherapy for primary brain cancer may be affected by a number of late effects, including hearing loss [10], [11], hair loss [11], brainstem injury or necroses [10], [12], [13], metabolic disorders, endocrine disorders, obesity and high BMI [14], abnormal early puberty [14], [15], [16], secondary cancer [10], vision loss [17], hypopituitarism [14], [15], [18], hypothyroidism [11], [16], diabetes insipidus [14], [16], other hormonal disorders [10], including growth hormone deficiency [11], [14], [15], [16], which results in small stature.

Radiotherapy incorporates both Proton Radiotherapy (PRT) and Photon Radiotherapy (XRT). PRT spares healthy tissue and has better accuracy than conventional radiotherapy [13], [19], [20], [21], [22], [23] and fewer late effects can be expected compared to XRT irradiation [23], [24]. Still, there is little knowledge of late effects as a direct consequence of PRT because of the numerous uncertainties in dose distribution, such as the impact of movement, linear energy transfer (LET) and relative biological effectiveness (RBE) [22]. PRT is a relatively new treatment where the first hospital-based facility was established in 1990 [22]. As of February 2024, there are 119 operating facilities worldwide [25].

Cancer as a disease is not just a medical understanding, with clear distinctions between body and mind. Having a biopsychosocial approach is important to understand the individual's vulnerability, social context, and health challenges [26], [27], [28].

**Fig. 1:**
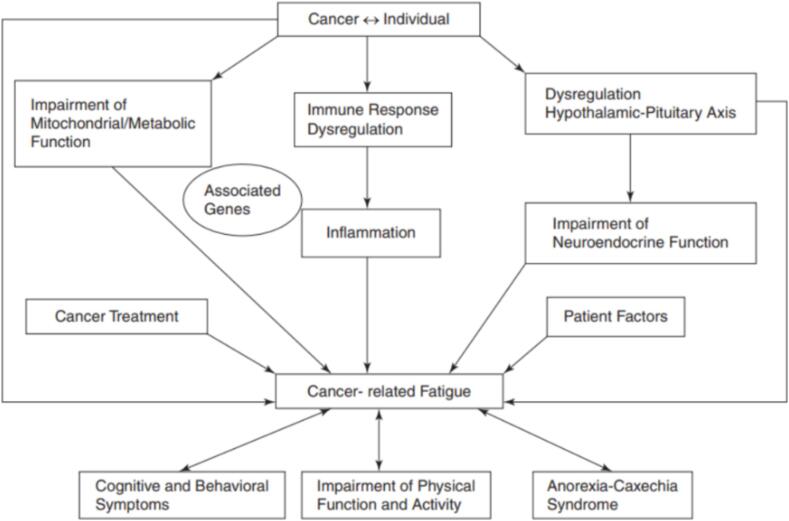
Influencing factors of CrF [29].

Cancer-related fatigue (CrF) can be experienced as distressing, and is a persistent, subjective feeling of exhaustion and fatigue, both physical, emotional and/or cognitive. This sensation may be related to cancer, or cancer treatment. It is not proportional to recent activity and will interfere with normal function. CrF is persistent and constant; lasting for more than 6 months, it will affect everyday life and not resolve with rest or sleep. We base our understanding of CrF on Gebauer's definition and figure. According to Fig. 1, CrF may both be caused by, and be a consequence of, cognitive and behavioural symptoms, impairment of physical function and activity and anorexia-cachexia syndrome [29]. These themes are repeated in the various themes found in the literature review.

WHO's definition of health from 1947 is formulated as “Health is a state of complete physical, mental and social well-being and not merely the absence of disease or infirmity” [30]. For a person with CrF, or essentially for all individuals, this might be unattainable. The individual may perceive their own health as good even if one does not meet the WHO's definition. Leonardi [31] considers the complexity children with CrF live with and focuses on the autonomous person. Good health includes the ability to react to events both physically and mentally, with a desired emotional or cognitive reaction [31]. In a modern definition of the concept of good health, the ability to be part of society in all aspects of life should be included [32]. It is Leonardi's and van Druten's understanding of the concept of health, with a personalized focus, on which we base this literature review.

Research has shown that cognitive outcomes following radiotherapy may be associated with fatigue [33], [34], [35], [36], [37]. Patients experiencing CrF may exhibit reduced cognitive function, leading to challenges in daily living, academic or work performance [36], [38], [39], [40]. Therefore, it may be relevant to consider fatigue as a potential factor influencing cognitive outcomes post-treatment.

The aim of this study is to investigate previous research on cognitive late effects that may be related to CrF in patients who have undergone primary brain radiotherapy before the age of 18.

## Methods

This systematic literature review was conducted based on the use of Preferred Reporting Items for Systematic Review and Meta-Analyses, PRISMA-S [41]. Literature searches were conducted in the databases MEDLINE ALL (Ovid), EMBASE (Ovid), CINAHL (EBSCO) and PsycINFO (Ovid). The search strategy was developed in cooperation with an information specialist and included medical subject headings and text words. The search strategy was similar for all databases. The search history from Ovid MEDLINE is shown in Table 1.

**Table 1:** Search history in Ovid Medline.

Ovid MEDLINE(R) ALL < 1946 to September 3, 2023>	
1	Exp Radiotherapy/
2	(radiotherapy or radiation therapy).tw.
3	1 or 2
4	Exp Brain/
5	(intracranial or cranial or brain).tw.
6	4 or 5
7	Exp Radiation Injuries/
8	(((side or late or adverse) adj2 effect*) or toxicit*).tw.
9	7 or 8
10	(child* or adolescen* or pediatr* or peadiatr* or youth).tw.
11	Adolecent/ or exp child/
12	10 or 11
13	3 and 6 and 9 and 12
14	Limit 13 to yr= ″2000−Current″

The last search was conducted 3rd September 2023. The search period was limited to 2000–2023 due to the implementation of modern treatment techniques such as IMRT, VMAT and PRT. This literature review focuses on malignant brain tumors. Inclusion and exclusion criteria are listed in Table 2 and were applied to sift through and exclude articles that were not relevant to our research focus.

**Table 2:** Inclusion and exclusion criteria.

	Inclusion criteria	Exclusion criteria
Types of studies	Primary studies that included multiple variations of the predetermined keywords for children, Radiation therapy, Brain, and late effects	Rewiever, Case report
Time frame	Published year 2000–2023	Treatment carried out before the year 2000
Focus	Studies that focused on children and young people under the age of 19 who undergo/have undergone radiatherapy for primary brain cancer	Studies that focused on chemotherapy or other drug treatment. Focus on metastases, secondary brain cancer, sarcomas, acute lymphoblastic leukemia (ALL), acute myelogenous leukemia (AML), benign tumorgroups. Focus on adults or both children and adults. Focus on side effects such as damage and malformation of blood vessels. Focus on acute side effects.
Information	Included information on radiotherapy (protons and photons), cognitive late effects, fatigue.	No specifies information on age of the pationts. No information on radiation dose. No information on follow-up

In this literature search, late effects were initially viewed from a broad perspective. It revealed trends related to patient challenges, especially cognitive ones. Few articles on fatigue as a late effect were found, leading to a targeted search with “fatigue” as the keyword. This search resulted in 264 records, which were narrowed down to three relevant articles focusing on CrF from treatment. However, these articles lacked detailed information on radiotherapy, treatment period, technique, and dosage, and were therefore excluded as a part of the main findings. Either way they were included as an additional finding, as they show a connection to the late effects found in the main search and therefore highlight the late effects as a relevance to CrF.

The references from the various databases were collected in an EndNote library [42], where duplicates were removed. The remaining records were transferred to Rayyan QCRI [43], where title and abstract screening was conducted. The authors worked in pairs, in a blinded process, by possessing different skills and knowledge. Conflicts were collectively reviewed to determine inclusion or exclusion. The authors screened the full-text articles in the same blinded process and assessed the quality of the included studies with checklist for cohort studies developed by the Joanna Briggs Institute (JBI) [44]. Braun and Clark's thematic analysis was used to summarise and analyse the data [45].

## Results

**Fig. 2:**
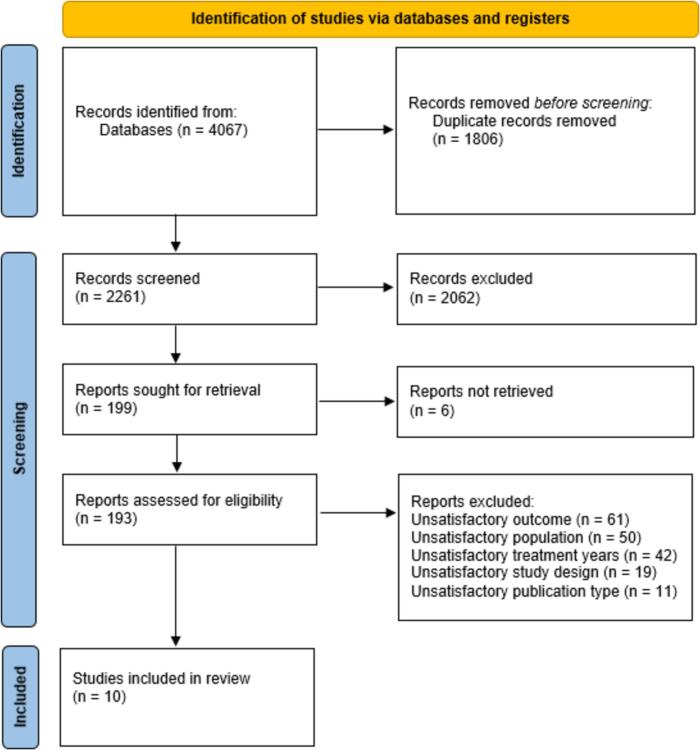
PRISMA flow chart of selection of studies [46].

A total of 4067 articles were identified through the database searches, 1806 duplicates were removed, and 2261 articles were screened by title and abstract. In total, 199 articles were reviewed in full text, of which 183 were excluded in the full text screening shown in Fig. 2, PRISMA flow chart. Finally, 10 studies were included. The four themes identified from the data were 1) neurocognitive functions and disorders, 2) intellectual functioning, 3) specific cognitive functions and 4) daily life, social functioning, and performance. These four themes can be embraced by the concept of CrF.

According to Gebaueŕs definition on CrF [29], the findings from this study may be related or connected to CrF. Three studies from the additional search show a direct context between some of the late effects in the main finding, and CrF.

### Characteristics of included studies

The literature matrix presents the included studies. All the studies were variants of cohort studies and included dosage information. All studies featured multimodal treatment regimens, encompassing chemotherapy [11], [21], [47], [48], [49], [50], [51], [52], [53], [54], with six of the studies incorporating surgical intervention [21], [47], [48], [49], [50], [54].

Seven studies were carried out in USA [47], [48], [49], [50], [52], [53], [54], two in Switzerland [11], [21] and one as a collaboration with Morocco, France and USA [51]. Seven studies included patients treated with tumors located in the infratentorium and/ or supratentorium [11], [21], [47], [50], [52], [53], [54] and three studies include patients with tumors located in the infratentorium/ posterior fossa only [48], [49], [51]. Three studies investigated patients treated exclusively with XRT [47], [50], [51], four with PRT [11], [21], [48], [54], and three with either XRT or PRT [49], [52], [53] (Table 3).

**Table 3:** Literature matrix.

Author Year	Country	Study design	Population Age	Treatment	Treatment period	Location	Follow-up Median (range)	Dosis- Gy (Range)	Cognitive test	Late effects finding	JBI rating
Ares et al 2016[11]	Switzerland	Retrospective cohort study	N=502.6 y (at RT)(1.1-15.2)	Pencil beam scanning- proton therapy (PBS-PT) and chemotherapy	2004-2013	Infratentorial 36 (72%) Supratentorial 14 (28%)	3.6 y (0.7-9.5)	59.4 Gy(54-60)1.8-2 fx	MRIs and CTCAE v4.0	1 (2%) patient had grade 1 concentration problem at follow-up	Good
Armstrong et al 2016[47]	USA	Retrospective, longitudinal cohort study	N=502.6 y (at RT)(1.1-15.2)	Photon radiation therapy (XRT),Chemotherapy and surgery	2006-2009	Infratentorial 13 (37%)Supratentorial 22 (63%)	Baseline 3.7m, 1y and 2y	56.7 Gy1.8 fx	RAVLT_T1-5, and ROCFT, and PictRecHR and PictRecRT	Verbal-semantic memory declined and was at its lowest measured at one year, but back to baseline at 2-years follow-up. Visual -perceptual memory were doble dissociated at baseline and 2 months. Recovery was found 2 years after XRT	Good
Grewal et al 2019 [48]	USA	Retrospective cohort study	N=143.3 y (at RT)(0.9- 5.2)	Proton therapy, chemotherapy, and surgery	2010-2017	Posterior fossa (100%)	4.5 y (0.3-6.9)	54 Gy1.8 fx	CTCAE-v.4.0	Some patients had initial decline in performance status, but all returned to baseline within two years after irradiation	Good
Kahalley et al 2020[49]	USA	Comparative, longitudinal study design	N=798.6 y (at DG)(3.5-15.3)	Photon radio- therapy (XRT), Proton radiotherapy (PRT), chemotherapy and surgery	2007-2018	Posterior fossa (100%)	4.3 y(0.1-10.9)	XRT: 55.8 Gy (54.0-59.4)PRT: 54 Gy (51-55.8)	WISC-V or WJ- III or SB5	4 years post-RT- PRT patient on average showed stable performance over time in all domains except processing speed. XRT patients exhibited a significant decline in global IQ, working memory and processing speed scores	Very good
Kahalley et al 2016[50]	USA	Retrospective cohort study	N=578.3 y (at RT)(1.7-14.6)	Cranial radiation therapy (RT), chemotherapy and surgery	NA	Infratentorial 26 (45.6%)Supratentorial 29 (50.9%) Both 2 (3.5%)	4.3 y(1.0-12.4)	54 Gy (45-60)	WISC-IV and Berry VMI	Surviving patients experience weaker cognitive proficiency than general reasoning ability. Performance on procession speed and working memory was particularly weak	Good
Khalil et al 2018[51]	Marocco, France and USA	Retrospective cohort study	N= 166.8 y (at DG)(4-11)	Radiation therapy (RT) and chemotherapy	2008-2012	Posterior fossa (100%)	4.0 y(3.0-5.0)	54 Gy + boost1.8 fx	WISC-IV	Significant impairment was found in at last one neurocognitive function; 88% decline in procession speed, 71% working memory, 68% verbal comprehension and 82% perceptual reasoning	Good
Mash et al 2023 [52]	USA	Retrospective cohort study	N=806.9 y (at DG)(0.8-17.9)	Conventional photon radiotherapy (XRT), Proton radiotherapy (PRT) and chemotherapy	XRT; 2000- 2007PRT; 2007- 2013	XRT;Supratentorial 11 (38%)Infratentorial 17 (59%)Both 1 (2%)PRT;Supratentorial 26 (51%)Infratentorial 24 (47%)Both 1 (3%)	>1 y following RT	XRT: 52.8 Gy (30.6-59.4)PRT:52.2 Gy (45-59.4)	WISC-V, WISC-IV or VAIS-IV	PTR patients demonstrated superior verbal learning and recall compared to XRT group. PRT group showed higher intellectual and adaptive function, and less concerns about day-to-day attention and cognitive regulation	Good
Mash et al 2023 [53]	USA	Longitudinal, observational study design	N=456.5 y (at DG)(0.8-16.1)	Photon radiotherapy (XRT), Proton radiotherapy (PRT) and chemotherapy	XRT: 2000-2007 PRT: 2007-2013	XRT;Supratentorial 4 (40%)Infratentorial 6 (60%)PRT;Supratentorial 6 (50%)Infratentorial 6 (50%)	>7 y past RT	XRT: 53.3 Gy (45-59.4)PRT: 53.5 Gy (53.5-59.4)	WISC-V, WISC-IV or VAIS-IV and VMI-VI	XRT group scored significantly lower than PRT group findings across all measures of cognitive and motor functioning, but there was no significant difference PRT and XRT group in any measure	Good
Tranet al 2020 [21]	Switzerland	Retrospective cohort study	N=2214.1 y (at RT)(0.8-18.2)	Pencil beam scanning proton therapy (PBS-PT), chemotherapy and surgery	1999-2017	Supratentorial 108 (49%)Infratentorial 100 (45.2%)Brainstem 13 (5.9%)	4.3 y(0.3- 12.0)	54 Gy (18.0-64.8)	CTCAE-v4.0. and PEDQOL	Cognition and social functioning scores were reported lower than norm at later time points than before PT. Typical late intellectual impairments and deficits in social adaption	Good
Zureichet al 2018 [54]	USA	Retrospective cohort study.	N=7012.1 y (at RT)(5.0-22.5)	Proton radiotherapy (PRT), chemotherapy and surgery	2002-2013	Supratentorial 34 (48.6%) Infratentorial 36 (51.4%)	3.0 y(1.1-11.4)	≤23.4Gy CSI 20 (57.1%),>23.4-Gy CSI 15 (42.9%), IF, involved field 32 (45.7%), Whole ventricle plus IF 3 (4.3%)	CMS and WMS. WISC-IV, WPPSI or VAIS-IV	Verbal memory were significantly declined at follow-up. But overall, the verbal and visual memory outcomes were within the normal range at follow-up	Good

Berry VMI (Beery-Buktenica Developmental Test of Visual-Motor Integration). CMS (Children’s Memory Scale). CTCAE (Common Terminology Criteria for Adverse Events). LANSKY (Lansky Play-Performance Scale). PEDQOL (Paediatric Quality of Life Inventory). PictRecHR (Picture Recognition Hit Rate). PictRecRT (Picture Recognition Reaction Time). RAVLT (Rey Auditory Verbal Learning Test). ROCFT (Rey-Osterrieth Complex Figure Test). SB (Stanford-Binet Intelligence Scales). WAIS (Wechsler Adult Intelligence Scales). WISC (Wechsler Intelligence Scales for Children). WJ (Woodcock- Johnson Test of Cognitive Abilities). WMS (Wechsler Memory Scale). WPPSI (Wechsler Preschool and Primary Scale of Intelligence). RT (Radiotherapy). PRT/PT (Proton Therapy). XRT (Photon radiation therapy). DG (diagnosis), Fx (fraction), Gy (Gray).

Curative-intent radiotherapy for children and young people with brain cancer follows standardized protocols and as such the radiotherapy doses were similar across studies. Radiotherapy for brain cancer with curative intent follows standardized protocols [55]. The period of follow-up varies, both in time of initiation, length, and interval between follow-up assessments. The studies used one or more neuropsychological tests designed to assess cognitive functions (Table 3). The two most commonly employed neurocognitive assessments were the WAIS, used in three different studies, and the WISC, which was used in six. The remaining tests were used once. For the patients in the studies performed by Tran, Ares and Gewal, the Common Terminology Criteria for Adverse Events (CTCAE) is used, rather than a formal neurocognitive test. The studies report various time points for initiation of cognitive testing, some before and others after treatment, and some not specified.

Table 4 provides an overview of three articles that investigated fatigue in this group [40], [56], [57]. The findings are reported retrospectively, post-radiotherapy. The treatment regimens for the included patients vary, but all patients received radiotherapy. The studies were conducted in Denmark, the USA, and Canada, respectively. The follow-up period ranges from 3 months to over 5 years. The studies do not include dosage data but are included to illustrate the association between reported fatigue as a late effect.

**Table 4:** Additional search.

Author Year	Country	Study design	Population Age	Treatment	Treatment period	Location	Follow-up Median (range)	Dosis- Gy (Range)	Cognitive test	Late effects finding	JBI rating
Helligsoe et al 2023 [56]	Denmark	Retrospective cohort study	N= 1619.1 y (at DG) (0.87- 15.9)	Whole-brain irradiation (XRT), Focal radiation (XRT), ChemotherapySurgery	N.AAssessment time2019- 2021	Supratentorial 89 (55 %)Infratentorial 81 (50 %)	>5 y post DG	NA	TMT-A, Coding, CCPT HRT, CCPT d′, CCPT omissions CCPT commissions, Digit span, HVLT-R total, HVLT-R delayed, HVLT-R recognition, COWAT Letter S COWAT Animals, TMT-B	Neurocognitive outcomes for survivors treated with surgery were below normative expectations. A number of survivors experienced significant fatigue (40%)	Good
Levitch et al 2022 [57]	USA	Retrospective cohort study	N= 42 (17 control)12.3 y (at DG) (6-16)	Surgery (100%)Photon radiotherapy (XRT) (64 %)	2015-2020	Infratentorial 25 (100 %)	4.3 y post DG	NA	Wechsler Intelligence Scale, California Verbal Learning Test, Beery-Buktenica Test, Grooved Pegboard or Purdue Pegboard, PedsQL Multidimensional Fatigue Scale	Processing speed difficulties were independent of fine motor functioning and fatigue, while better intellectual functioning, working memory, verbal memory recall, and visual-motor integration abilities were associated with higher parental education	Good
Macartney et al 2014 [40]	Canada	Observational cross-sectional study (preformed post-RT)	N= 506.4 y (at DG) (3.0-11.6)	Photon radiotherapy (XRT), Surgery, chemotherapy	N.A Assessment time 2011-2012	Supratentorial 25 (50 %)Infratentorial 25 (50 %)	>3 months	NA	MSASPedsQL	Lack of energy or fatigue was identifiedas a prevalent, severe, and distressing symptomin paediatric brain tumour survivors	Good

### Neurocognitive functions and disorders

It is shown that PRT may be favourable in preserving cognitive function compared to XRT therapy, thus reducing neurocognitive late effects [52]. Neurological late effects among cancer survivors are particularly linked to processing and working memory, necessitating targeted interventions [50], [51]. Although cognitive benefits of PRT have been shown [52], research shows impaired processing and working memory on quality of life and academic performance in survivors of radiotherapy [50].

### Intellectual functioning

Identified key medical risk factors included time since radiotherapy, chemotherapy, tumor location and diagnosis of medulloblastoma. Total tumor dose emerged as a significant factor influencing IQ scores [50]. PRT were related to stable IQ, in contrast to significant decline observed in XRT recipients. Despite similar average global IQ scores, the PRT group remained stable since diagnosis, while the XRT group experienced an annual decline [49]. Age influences cognitive outcomes and achievements. Younger patients show lower scores on cognitive tests [51].

### Specific cognitive functions

The studies in this review, the specific cognitive function results of paediatric brain tumor patients show variation across different treatment modalities. While one study observed minimal concentration problems post-treatment [11], another showed fluctuations in verbal memory over time [47]. A correlation was identified between radiation to the left hippocampus and decline in verbal memory, particularly pronounced in women [54]. Comparable verbal reasoning abilities were found in both PRT and XRT-group [49], while superior verbal memory was shown in PRT-group [52]. Stable perceptual reasoning were highlighted in the PRT-group [49].

### Daily life, social functioning and performance

The findings suggest that paediatric patients may experience temporary declines in performance status following radiotherapy, with most cases returning to baseline within two years [48]. Treatment-related factors such as age at radiation and dose are predictors of neurological challenges, especially among medulloblastoma survivors [51]. One study notes participants’ cognitive and social interaction problems after PRT [21], while another reports successful school reintegration for the majority, with only a minority needing special support [48]. These results underscore the complex challenges facing paediatric cancer survivors' aftercare, necessitating tailored interventions to address cognitive and socio-emotional needs.

### Cancer related fatigue

Research has shown that CrF can relate to cognitive late effects after cranial irradiation, highlighting a correlation between verbal fluency, sustained attention, working memory, processing speed, concentration, and fatigue as shown in Table 4 [40], [56], [57]. The studies showed that the more fatigue individuals experience, the more their cognitive function, such as working memory, is impacted [56], [57]. The same is also shown in relation to processing speed, where an increased experience of fatigue leads to decreasing processing speed [56], [57]. Also, a correlation between more concentration problems was shown with increasing fatigue, and radiation entails a risk factor for fatigue among other effects [40].

## Discussion

This literature review summarises research on cognitive late effects that can be related to CrF for children treated with radiotherapy for primary brain cancer. Due to well-documented cognitive difficulties following radiotherapy [58], [59], [60], this study presents a synthesised outcome from multiple studies on the extent of CrF following treatment for primary brain cancer. Previous research demonstrates challenges across various domains including intellectual, neurocognitive, memory, comprehension, concentration, activities of daily living, and academic or occupational performance [40], [56], [61].

This literature review findings reveal variations in cognitive outcomes after radiotherapy, with both PRT and XRT showing distinct effects. While PRT patients show stable performance and superior verbal learning compared to XRT, XRT patients experience significant reductions in global IQ, working memory, and processing speed. Despite these differences, both groups show poorer cognitive ability relative to general reasoning, underscoring the intricate nature of post-treatment cognitive outcomes [11], [21], [47], [48], [49], [50], [51], [52], [53], [54].

The findings underscore the significant impact of radiotherapy on individuals’ post-treatment. Some of the included studies observed that treatment with PRT may potentially reduce neurocognitive late effects, which emphasizes the importance of treatment choices to minimize long-term cognitive damage [52]. Furthermore, two studies point out that cancer survivors experience significant neurological late effects, especially processing speed, verbal memory and working memory [50], [51]. These factors are crucial for children's learning and daily function [62]. The findings show that children who receive radiotherapy, especially XRT, experience significant decreases in these areas compared to PRT or control groups [50], [53].

Difference between treatment with XRT and PRT are also highlighted by Kahalley at al. [49], where the XRT-group showed significant decrease in global IQ [49]. Several studies showed a predominant benefit from PRT in the preservation of verbal memory, working memory and perceptual reasoning [11], [47], [49], [52], [54]. This difference in cognitive function between the treatment groups underlines the importance of individualized treatment strategies [63]. Although it appears that PRT can be linked to less cognitive decline [49], [52], [53], it is important to problematize the follow-up with the various treatment methods and access that is variable. The time-period for follow-up may have impacted the results of the studies. Only one study provides a clear description on different test registration times and includes a detailed baseline [47]. It is challenging to draw definitive conclusions given the substantial variations present. From a radiotherapeutic perspective, it is difficult to highlight clear treatment-related consequences correlating to radiotherapy and cognitive decline. The studies do not provide clear information on timing of testing and data collection. One can debate whether it is possible to draw a line between cognitive changes, CrF and radiation therapy without pre-treatment tests.

Studies incorporated in this literature review illuminate the significance risk of medical factors linked to cognitive challenges. Kahally at al. [50] highlight the complexity of multimodal treatment, timing, tumor location, specific diagnoses and intellectually outcomes [50]. The studies used different test methods, treatment regimens, testing times, and even the specific target group had many variables. This makes it difficult to draw clear and generalised conclusions when comparing different studies [64]. Age at diagnosis is an important factor in relation to cognitive outcomes [51]. Younger children are particularly vulnerable, regarding the comprehensive learning requirements in kindergarten and school. The social functioning and everyday life of these children can be negatively affected [21], [51], including social isolation [65] and challenges in school performance [66], which may impact their quality of life. This emphasizes the importance of social support and adaptations in the learning environment to meet every child’s unique needs [67], [68].

The results presented in this study emphasize the wide range of cognitive challenges these children face. The results indicate that although some children show signs of improvement in performance status over time, there are clear cognitive and social challenges that affect their ability to function optimally in everyday and social settings. For some, these challenges may be burdensome and exhausting [69].

A reduction in cognitive function may lead to greater mental fatigue in both daily and academic activities [70]. A relationship is also shown between CrF, processing speed, working memory and performance [71]. This can potentially increase the mental load and the degree of exhaustion, tiredness, and fatigue [72]. Although fatigue and CrF are not directly mentioned or discussed in the studies as a late effect, even low degrees of late effects can be indirectly linked to CrF [40], [56], [57]. It emphasizes the need for a holistic approach to treatment and rehabilitation. Both the cognitive and socio-emotional aspects are essential to manage CrF effectively.

These findings underscore the need for better understanding and targeted interventions to address the long-term cognitive sequelae in children undergoing radiotherapy. As of today, a randomized controlled trial, PRO-GLIO, investigates fatigue as a late effect after brain tumor PRT. Even though they include patients aged 18–65, this is an important step in the mapping of fatigue after radiotherapy to the brain [73].

During the investigation of existing literature for former paediatric brain tumor patients struggling with fatigue, several studies discussed fatigue as a direct consequence of radiotherapy, without mentioning either dose or technique [40], [56], [57]. Radiation dosage as a factor to understand neurocognitive challenges is crucial [50], [51].

Even though all the included studies give information about the integral dose, just a few provide information about dose per fraction [11], [47], [48], [51]. At the same time, only one study provided specific information about organ dose [47]. It might have been useful to know e.g., what percentage of the brain received 50 % of the maximum dose or what area received 20 % of the maximum dose and the dosage given to the organ at risk. Prior studies have shown that patients receiving a dose directly to the hippocampus has a decline in neurocognitive functions after cranial radiotherapy [74], [75]. Previous research has demonstrated that when attempting to draw conclusions about potential associations between radiotherapy and side effects that information about total dose, dose per fraction andvolume of the brain irradiated are useful. For example, one study showed that a dose of 18.1 Gy to 100 % of the brain results in a 5 % risk of IQ < 85 [60].

### Strengths and Limitations

The study ensures replicability through adherence to PRISMA-S guidelines. Searches were conducted in multiple medical databases, and a rigorous blinded screening and critical appraisal of the studies were conducted. The explicit presentation inclusion and exclusion criteria of the study, complemented by a detailed explanation of the rationale behind their selection, adds integrity to the study. Our pre-existing practical knowledge of the subject matter and professional terminology is a significant advantage conducting this study.

The included studies vary in terms of population, follow-up, treatment, treatment period, dosage and cognitive tests. These factors will influence the results in this literature review. The data material includes treatment with both XRT and PRT and varying follow-up which may be natural considering PRT is a relatively new treatment. The variation in the length, and intervals of the follow-up, may make general comparison difficult.

There were different cognitive tests. Nevertheless, it must be considered that the clinics have chosen the tests they believe are most accurate and appropriate, as well as practical to administrate at the time.

It is challenging to associate cognitive changes directly to radiotherapy without access to precise information about the timing of testing, or whether pre-treatment testing was performed to provide a baseline.

Due to the universal use of chemotherapy and surgical interventions, it is challenging to conclusively attribute the cognitive challenges as late effects solely to radiotherapy. One of the exclusion criteria, lack of dose information, was a possible unnecessary limitation regarding the studies’ small range in dose. Not including this limitation could have allowed inclusion of a greater number of studies. On the other hand, it is challenging to generalize the effect dose has on children's cognition without dose information. This lack of knowledge should be further researched.

## Conclusion

The research here conducted provide crucial insights into previous research on cognitive late effects that can be related to CrF in patients who have undergone primary brain radiotherapy before the age of 18. In our society, as we move through different social environments, the inability to fully use onés skills and potential can lead to negative health outcomes. We acknowledge the need for more information on both proton and photon irradiation for these patients. Although some children have received proton therapy, it has become evident that they too experience cases of late effects and fatigue.

Having detailed information on specific treatments, disease progression, target volume size, and doses to surrounding organs at risk is crucial. This knowledge is vital for improving outcomes in affected children and underscores the importance of addressing and reducing late side effects, such as fatigue.

## Declaration of competing interest

The authors declare that they have no known competing financial interests or personal relationships that could have appeared to influence the work reported in this paper.
